# AMBRA1-Mediated Mitophagy Counteracts Oxidative Stress and Apoptosis Induced by Neurotoxicity in Human Neuroblastoma SH-SY5Y Cells

**DOI:** 10.3389/fncel.2018.00092

**Published:** 2018-04-18

**Authors:** Anthea Di Rita, Pasquale D’Acunzo, Luca Simula, Silvia Campello, Flavie Strappazzon, Francesco Cecconi

**Affiliations:** ^1^IRCCS Fondazione Santa Lucia, Rome, Italy; ^2^Department of Biology, University of Rome Tor Vergata, Rome, Italy; ^3^Department of Paediatric Haematology and Oncology, IRCCS Bambino Gesù Children’s Hospital, Rome, Italy; ^4^Unit of Cell Stress and Survival, Danish Cancer Society Research Center, Copenhagen, Denmark

**Keywords:** cell death, oxidative stress, *in vitro* models, Parkinson’s disease, mitophagy

## Abstract

Therapeutic strategies are needed to protect dopaminergic neurons in Parkinson’s disease (PD) patients. Oxidative stress caused by dopamine may play an important role in PD pathogenesis. Selective autophagy of mitochondria (mitophagy), mainly regulated by PINK1 and PARKIN, plays an important role in the maintenance of cell homeostasis. Mutations in those genes cause accumulation of damaged mitochondria, leading to nigral degeneration and early-onset PD. AMBRA1^ActA^ is a fusion protein specifically expressed at the mitochondria, and whose expression has been shown to induce a powerful mitophagy in mammalian cells. Most importantly, the pro-autophagy factor AMBRA1 is sufficient to restore mitophagy in fibroblasts of PD patients carrying PINK1 and PARKIN mutations. In this study, we investigated the potential neuroprotective effect of AMBRA1-induced mitophagy against 6-hydroxydopamine (6-OHDA)- and rotenone-induced cell death in human neuroblastoma SH-SY5Y cells. We demonstrated that AMBRA1^ActA^ overexpression was sufficient to induce mitochondrial clearance in SH-SY5Y cells. We found that apoptosis induced by 6-OHDA and rotenone was reversed by AMBRA1-induced mitophagy. Finally, transfection of SH-SY5Y cells with a vector encoding AMBRA1^ActA^ significantly reduced 6-OHDA and rotenone-induced generation of reactive oxygen species (ROS). Altogether, our results indicate that AMBRA1^ActA^ is able to induce mitophagy in SH-SY5Y cells in order to suppress oxidative stress and apoptosis induced by both 6-OHDA and rotenone. These results strongly suggest that AMBRA1 may have promising neuroprotective properties with an important role in limiting ROS-induced dopaminergic cell death, and the utmost potential to prevent PD or other neurodegenerative diseases associated with mitochondrial oxidative stress.

## Introduction

Parkinson’s disease (PD) is a chronic and severe neurodegenerative disorder characterized by a progressive and selective death of dopaminergic neurons in the substantia nigra. The cellular loss (among 50%–70%) in the ventral area of the substantia nigra pars compacta is the main pathological hallmark of PD. Although Parkinsonism is usually a sporadic disease, there is also a familial component related to a growing number of single gene mutations. Among these, mutations in mitochondrial genes encoding PINK1, PARKIN and DJ-1 proteins promote autosomal recessive PD. Although the exact causes defining PD are largely unknown, mitochondrial oxidative stress and accumulated dysfunctional mitochondria are critical factors in the disease onset. The clearance of dysfunctional mitochondria can be regulated by a selective form of autophagy, known as mitophagy (Lemasters, 2005). Mitophagy is a self-degradative process that allows the elimination of ubiquitin-targeted mitochondria through lysosomal digestion. Both PINK1 and PARKIN are key factors in mitophagy induction: PINK1 is a Ser-Thr kinase that mediates the phospho-ubiquitin signal, thus recruiting the E3 ubiquitin ligase PARKIN at the mitochondria. Once at the mitochondria, PARKIN amplifies the ubiquitin signal on the mitochondrial surface, this leading to their recruitment into the autophagosome (Lazarou et al., 2015). We have previously demonstrated that a mitochondria-targeted fusion protein, AMBRA1^ActA^, is a novel powerful inducer of mitophagy in mammalian cells (Strappazzon et al., 2015). Most importantly, we demonstrated that AMBRA1 restores mitophagy in mouse embryonic fibroblasts from *PINK1^−/−^* mice, but also in fibroblasts from PD patients, in which PINK1 and PARKIN were mutated; altogether, these findings highlighted AMBRA1 as an alternative mediator of mitophagy to maintain mitochondrial homeostasis in PINK1-PARKIN-related PD (Strappazzon et al., 2015). In the PD context, oxidative stress is widely considered to be a key factor in both familial and sporadic forms of the disease (Sanders et al., 2014). It results from an imbalance of pro-oxidants/anti-oxidants homeostasis that leads to an abnormal production of reactive oxygen species (ROS), whose overproduction generates damage of both neurons and astrocytes (Lin and Beal, 2006).

In this study, we investigated the effect of AMBRA1^ActA^ protein in two oxidative stress models, evoked by disruption of the mitochondrial activity induced by: (1) blockade of mitochondrial complexes I and IV by using the pro-oxidant derivate of dopamine, 6-hydroxydopamine (6-OHDA; Glinka et al., 1997); or (2) blockade of mitochondrial complex I by using rotenone, a pesticide that has been associated with increased risk for PD (Li et al., 2003; Chin-Chan et al., 2015) in dopaminergic neural SH-SY5Y cells (Van Humbeeck et al., 2011). We further show that AMBRA1^ActA^ expression is sufficient to induce mitophagy also in SH-SY5Y cells. Moreover, the induction of mitophagy preserved cells from apoptosis induced by 6-OHDA and rotenone. Indeed, we observed an increase of cell viability in cells positive for AMBRA^ActA^, associated with a reduction of PARP-1 cleavage (caspase-3 substrate) and a number of pyknotic nuclei. Finally, transfection of SH-SY5Y cells with a vector encoding AMBRA1^ActA^ significantly reduced 6-OHDA- and rotenone-induced generation of ROS. Our results thus indicate that AMBRA1^ActA^ is able to induce mitophagy in SH-SY5Y cells to suppress oxidative stress and apoptosis induced by 6-OHDA and rotenone. This evidence proves that AMBRA1^ActA^ may represent a novel molecular tool that could be used to induce mitophagy, in order to prevent accumulation of damaged mitochondria and neurodegeneration in PD.

## Materials and Methods

### Cell Culture

Human neuroblastoma SH-SY5Y cells were cultured in Dulbecco’s modified Eagle’s medium (DMEM, GIBCO) supplemented with 10% FBS (GIBCO), at 37°C in a humidified atmosphere of 5% CO_2_.

### Reagents

3-(4,5-dimethylthiazol-2-yl)-5-(3-carboxymethoxyphenyl)-2-(4- sulfophenyl)-2H-tetrazolium (MTS) was purchased from Promega. 6-hydroxydopamine (6-OHDA), rotenone, the lysosome inhibitor NH_4_Cl and the Carbonyl cyanide 4-(trifluoromethoxy)phenylhydrazone (FCCP) were purchased from Sigma Aldrich.

### Transient Transfection and Plasmids

Human neuroblastoma SH-SY5Y cells were transfected using *Trans*IT-X2^®^ Dynamic Delivery System (MIR 6003, Mirus) according to the standard protocol. The Myc-AMBRA1^ActA^ plasmid was cloned as described in Strappazzon et al. (2015). The Mito-DsRED construct encodes for human Cox8A mitochondria signal peptide which is fused with wild-type-DsRED in PcDNA3 vector (Invitrogen). The Mito-DsRED-AMBRA1^ActA^ is a bidirectional vector encoding for both Mito-DsRED and AMBRA1^ActA^. For small interfering RNA (siRNA)-mediated knockdown of AMBRA1, siRNA against AMBRA1 or siRNA-Ctr (Invitrogene, were transiently transfected using Lipofectamine 2000, according to the manufacturer’s protocol. ShRNA PARKIN or shRNA Control were transfected in SH-SY5Ycells using Turbofect Transfection reagent (Thermo Fisher Scientific, R0531), according to the standard instructions.

### Antibodies

We use the following antibodies: anti-Myc (Santa Cruz Biotechnology, 9E10), anti-ACTIN (Sigma-Aldrich, A2228), anti-PARP (Cell Signaling, 9542), anti-MnSOD (Enzo Life Sciences, 110F), anti-TOM20 (Santa Cruz Biotechnology, sc-FL145) and anti-HSP60 (Santa Cruz Biotechnology, sc-13966).

### Immunoblotting Analysis

SH-SY5Y cells were lysed in RIPA buffer (50 mM Tris HCl pH 7.4, 1% Triton X-100, 0.5% NP40, 150 mM NaCl, 10% Glycerol, 2.5% Sodium Deoxycholate) plus protease inhibitor cocktail (Sigma Aldrich, S7920) and analyzed by SDS/PAGE and Western blot according to standard protocols.

### Immunofluorescence Analysis

SH-SY5Y cells were fixed in 4% of paraformaldehyde and permeabilized with 0.4% Triton X-100. Fixed cells were blocked in 2% normal goat serum (Sigma-Aldrich, G9023) in PBS and incubated with primary antibody over night. Cells were then washed with PBS 3 × 5 min and incubated with secondary antibody for 1 h. After 3 × 5 min of PBS washing, coverslips were mounted on glass slides and finally analyzed by confocal microscopy analysis under Zeiss LSM 700 100× oil- immersion objective (CLSM700; Jena, Germany), Zoe Fluorescence cell imager (Biorad, 1450031) or Ultraview Vox (Perkin Elmer).

### Confocal Image Analysis

The TOM20 and HSP60 intensities and the area occupied by the signal per cell, in conditions indicated in the text, were calculated through the NIH ImageJ software in 10 different fields of three independent experiments. Colocalization measurements were made through the JACOP plugin (Bolte and Cordelières, 2006) of the NIH ImageJ software. M1 manders colocalization coefficients (MCC) of mitochondria overlapping LC3, p62 and Ubiquitin were calculated on single cells in 10 different fields of three independent experiments upon overexpression of either Mito-DsRED or Mito-DsRED -AMBRA1^ActA^ to highlight wild type mitochondria and mitoaggresomes, respectively. Thresholds were not set by the operator, but automatically calculated by the software to avoid biased data. All microscope quantifications shown in the article were performed by a blind approach.

### Determination of Cell Viability

Cell vitality was estimated by the MTS assay or by counting the number of condensed or fragmented nuclei (DAPI staining). For the MTS assay, the cytotoxicity of 6-OHDA or rotenone on SH-SY5Y cells was determined using the CellTiter96 Aqueous One Solution Assay (Promega, Madison, WI, USA). Indeed, SH-SY5Y cells were transfected using *Trans*IT-X2^®^ Dynamic Delivery System (MIR 6003, Mirus). Six hours after transfection, cells were split into 96-well dishes and treated with 6-OHDA (100 μM) or rotenone (10 μM). After 18 h, the CellTiter 96 AQueous One Solution Reagent was added to each well. The plate was then read at 490 nm in a Victor^3^ plate reader at 37°C (PerkinElmer Life and Analytical Sciences, Shelton, CT, USA). For pyknotic nuclei counting, Mito-DsRED or Mito-DsRED-AMBRA1^ActA^ transfected cells were fixed in 4% paraformaldehyde (Merck Millipore, 104005) in PBS (GIBCO, BE17-512F) for 10 min at 37°C and stained with DAPI (Sigma Aldrich, D9542) in PBS for 15 min at room temperature. Cells were washed three times with PBS, then mounted and observed under Zoe microscope. For each condition, random images were captured and cell viability was then scored on the basis of nuclear morphology: cells containing condensed or fragmented nuclei were counted as dying or dead cells. All microscope quantifications shown in the article were performed by a blind approach.

### Protein Oxidation Assay

SH-SY5Y cells were transfected and treated with 6-OHDA or rotenone, as described above. The DPN-derivatized proteins containing carbonyl groups were evaluated by using the OxyBlot Protein Oxidation Detection Kit (Millipore S7150), according to the standard protocol.

### Measure of ΔΨm and Superoxide Production

Cells were transfected with the bicistronic vector Tween-GFP-AMBRA1^ActA^ or Twen-GFP alone and subsequently treated or not with 100 μM 6-OHDA and 10 μM rotenone 6 h post transfection. After 18 h of treatment, cells were incubated with 5 nM Tetramethylrhodamine methyl ester (TMRM, Molecular Probes) in 10% FBS+DMEM for 30 min at 37°C or with 5 μM MitoSOX Red reagent (Molecular Probes) in 10% FBS+DMEM for 10 min at 37°C in a 5% CO_2_ incubator. Finally, cells were analyzed through the flow cytometer BD Accuri C6. TMRM and MitoSOX Red fluorescence intensities were measured in GFP positive cells and read in the FL3 channel upon stimulation at 488 nm.

### Statistical Analysis

All statistical analysis were performed and graphed using GraphPad Prism 6. Comparisons between two groups were analyzed using Student’s *T*-test. Three or more groups comparisons were performed with one-way ANOVA. Significance is defined as *P < 0.05.

## Results

### High Levels of Mitochondria-Targeted AMBRA1 (AMBRA1^ActA^) in Human Neuroblastoma SH-SY5Y Cells Induce Perinuclear Distribution of Mitochondria, Accompanied by Mitochondria Clearance

**Figure 1:**
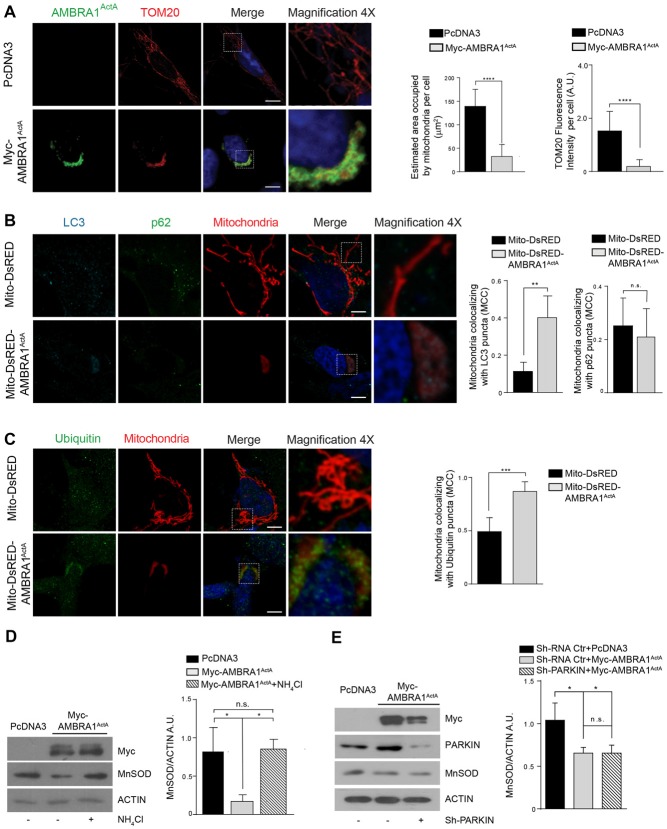
AMBRA1^ActA^ induces mitophagy in SH-SY5Y cell line. **(A)** SH-SY5Y cells transfected with PcDNA3 or Myc-AMBRA1^ActA^ were immunostained with anti-Myc (green) and anti-TOM20 (red) antibodies. Scale bar, 10 μm. The upper and lower panels on the right are two magnifications (4×) of the mitochondria in transfected cells. The graphs show the estimated area occupied by mitochondria per cell and the TOM20 fluorescence intensity per cell (± S.D). *n* = 3 independent experiments. Statistical analysis was performed using Student’s *T*-test. ****P < 0.0001. **(B)** SH-SY5Y cells were transfected with vectors encoding Mito-DsRED or Mito-DsRED-AMBRA1^ActA^ in order to mark wild type mitochondria and mitoaggresomes, respectively. Subsequently, cells were fixed and immunostained for the autophagosome marker LC3 (blue), the mitophagy receptor p62 (green) and counterstained with DAPI (blue). The graphs summarize the quantification in single cells of the manders colocalization coefficient (MCC) of mitochondria on the LC3 signal (left) or p62 signal (right). The upper and lower panels on the right are two magnifications (4×) of the mitochondria in transfected cells. Ten random fields of three independent experiments were considered. Data are presented as Mean ± S.D. Statistical test: student *T*-test. Scale bar: 5 μm. **P < 0.01; n.s. not statistically significant. **(C)** SH-SY5Y cells transfected as described in **(B)**, were immunostained for Ubiquitin (green) and counterstained with DAPI (blue). The graph shows the quantification in single cells of the MCC of mitochondria on the Ub signal. The upper and lower panels on the right are two magnifications (4×) of the mitochondria in transfected cells. Ten random fields of three independent experiments were considered. Data are presented as Mean ± S.D. Statistical test: student *T*-test. Scale bar: 5 μm. ***P < 0.001. **(D)** PcDNA3 or Myc-AMBRA1^ActA^ transfected cells, treated with the lysosome inhibitor NH_4_Cl, were analyzed by western blotting analysis for the indicated antibody. The graph shows the MnSOD/ACTIN *ratio*. Statistical analysis was performed using One-way ANOVA. *P < 0.05; n.s. not statistically significant. *n* = 3 independent experiments. **(E)** SH-SY5Y cells were transfected with Sh-PARKIN or Sh-RNA Ctr in combination with a vector coding for Myc-AMBRA1^ActA^. Total lysates were subjected to western blotting analysis using anti-MnSOD (to analyze mitochondrial clearance), anti-Myc or anti-PARKIN to assess Myc-AMBRA1^ActA^ or Sh-PARKIN transfections, respectively. The quantification represents the MnSOD/ACTIN *ratio*. Statistical analysis was performed using One-way ANOVA. *P < 0.05; n.s. not statistically significant. *n* = 3 independent experiments.

To manipulate the dosage of the AMBRA1 mitochondrial pool in order to induce massive mitophagy, we previously generated a fusion construct encoding Myc-AMBRA1 and a sequence from the *Listeria monocytogenes* Actin assembly-inducing protein (ActA), that can target the molecule to the outer mitochondrial membrane (Strappazzon et al., 2015). In this case, we wanted to check for the localization of the AMBRA1^ActA^ protein and for a putative mitochondrial clearance in SH-SY5Y cells. To this end, we performed a confocal microscopy analysis in SH-SY5Y cells transfected with a vector encoding PcDNA3 as a control or Myc-AMBRA1^ActA^. We then fixed cells and stained their mitochondria network by using an antibody directed against TOM20. We found that AMBRA1^ActA^ surrounds aggregated mitochondria. Most importantly, we observed that AMBRA1^ActA^ overexpression induces *per se* a strong relocalization of the mitochondrial network around the perinuclear envelope, thus leading to the formation of structures similar to those previously described as “mito-aggresomes” (Lee et al., 2010; Figure 1A). The reduction of both the area of mitochondria and the TOM20 signal intensity in AMBRA1^ActA^-transfected cells confirmed that AMBRA1^ActA^ stimulates massive mitophagy also in SH-SY5Y cells. Moreover, in order to ascertain that mito-aggresome structures were specific for AMBRA1 mitochondrial localization coupled with a reduction of mitochondrial marker levels, we performed a confocal microscope analysis, in which SH-SY5Y cells were transfected with PcDNA3, Myc-AMBRA1^ActA^, Venus^ActA^, a plasmid encoding the ActA sequence fused with the fluorescent reporter protein Venus (Nagai et al., 2002), and with the wild-type form of AMBRA1 lacking the ActA sequence. Further, the AMBRA1^ActA-LIRAA^ plasmid, encoding an AMBRA1^ActA^ isoform mutated in its LIR motif and less active in mitophagy (due to a reduction of LC3B binding), was used to complete this analysis (Strappazzon et al., 2015; Supplementary Figure S1). We next analyzed whether AMBRA1^ActA^ expression was able to induce mitophagy, as we already described in HEK293, HeLa and MEF cells (Strappazzon et al., 2015). To this end, SH-SY5Y cells were transfected with a bicistronic vector coding for Mito-DsRED alone (red staining of mitochondria) or encoding both Mito-DsRED and AMBRA1^ActA^ (Mito-DsRED-AMBRA1^ActA^) for 24 h; first, we analyzed mitophagy induction by looking to well-known markers of autophagosome formation, such as LC3 and p62 (Kabeya et al., 2000; Pankiv et al., 2007). Indeed, we observed an increase in the accumulation of LC3 dots, but not p62, in Mito-DsRED-AMBRA1^ActA^-positive mitochondria, suggesting that AMBRA1^ActA^ promotes an LC3-dependent, but p62-independent mitophagy in SHSY5Y cells (Figure 1B). In addition, we checked for ubiquitin staining and found that mito-aggresomes are positive for Ubiquitin (Figure 1C). These data proved that AMBRA1^ActA^ induces mito-aggresomes positive for LC3 and Ubiquitin, but negative for p62 also in SHSY5Y cells. We next confirmed that AMBRA1^ActA^ was able to induce mitophagy in these cells, by using a biochemical approach. Indeed, we checked the level of MnSOD, a mitochondrial protein, following AMBRA1^ActA^ transfection in SH-SY5Y cells in the presence or absence of a lysosomal inhibitor, ammonium chloride (NH_4_Cl), known to prevent the autophagy flux by inhibiting the fusion of autophagosomes with lysosomes (Hart and Young, 1991). As expected, AMBRA1^ActA^ was able to induce a strong reduction of MnSOD protein levels. In addition, the NH_4_Cl treatment resulted in a significantly higher level of mitochondrial protein accumulation in AMBRA1^ActA^-positive cells, hence indicating that autophagosome formation induced by AMBRA1^ActA^ overexpression is responsible for MnSOD degradation by the lysosome (Figure 1D). The ability of AMBRA1^ActA^ to mediate mitophagy in these cells was confimed also by looking at another mitochondrial marker, COXIV (data not shown). Finally, we checked in SH-SY5Y cells downregulated for PARKIN expression whether AMBRA1^ActA^ was able to induce mitophagy. As shown in Figure 1E, AMBRA1^ActA^ induces mitophagy also in PARKIN-deficient SH-SY5Y cells.

Altogether, these observations demonstrate that AMBRA1^ActA^ transfection triggers *per se* a fully functional and massive mitophagy in SH-SY5Y cells, which is independent of both PINK1/PARKIN and p62, but dependent of LC3 and Ubiquitin.

### AMBRA1^ActA^-Induced Mitophagy Protects SH-SY5Y Cells From Neurotoxicity Induced by 6-OHDA

**Figure 2:**
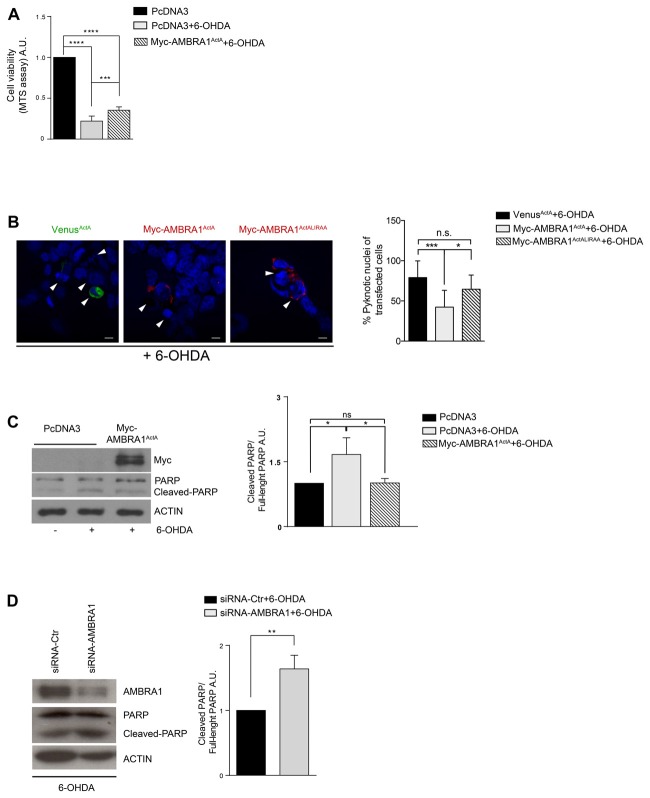
AMBRA1^ActA^ improves vitality of SH-SY5Y cells treated with 6-hydroxydopamine (6-OHDA). **(A)** SH-SY5Y cells were transfected with vectors coding for PcDNA3 or for Myc-AMBRA1^ActA^ and 6 h after transfection, cells were treated with 6-OHDA (100 μM). Viable cells were estimated using the MTS assay. Results are expressed as A.U. Each point represents the mean (± SD) of triplicate wells from three independent experiments. Statistical analysis was performed using One-way ANOVA. ****P < 0.0001; ***P < 0.001. **(B)** SH-SY5Y cells were transfected with vectors encoding Venus^ActA^ or Myc-AMBRA1^ActA^ or Myc-AMBRA1^ActALIRAA^ plasmids for 24 h. Six hours after transfection, cells were treated with 6-OHDA (100 μM). Cells were then fixed and stained with DAPI. Cells with condensed or fragmented nuclei were scored as pyknotic (arrows indicate pyknotic nuclei). The graph shows the percentage of pyknotic nuclei in transfected cells. For each condition, transfected cells were counted in random fields from three independent experiments. Statistical analysis was performed using One way ANOVA. ***P < 0.001; *P < 0.05; n.s. not statistically significant. **(C)** SH-SY5Y cells were transfected with vectors encoding PcDNA3 or Myc-AMBRA1^ActA^ for 24 h. Six hours after transfection, cells were treated with 6-OHDA (100 μM). After proteins extraction, we performed a western blot analysis using antibodies directed against AMBRA1 (Myc), PARP and ACTIN. The graph shows the cleaved PARP/Full-length PARP *ratio* resulting as the mean of three independent experiments (± S.D). *n* = 3. Statistical analysis was performed using One-way ANOVA, *P < 0.05. **(D)** SHSY5Y cells transfected with a siRNA-Ctr or siRNA-AMBRA1 were treated with 6-OHDA for 18 h. Total lysates were immunoblotted for AMBRA1, PARP and ACTIN antibodies. Statistical analysis was performed using One-way ANOVA. **P < 0.01. *n* = 3 independent experiments.

We have previously demonstrated that AMBRA1-induced mitophagy is able to trigger mitophagy in fibroblasts from PD patients bearing PINK1 or PARKIN mutations, this highlighting the fact that AMBRA1 can serve as an alternative mediator of mitophagy to maintain mitochondrial turnover in PINK1/PARKIN-related PD. Thus, since mitophagy plays an important role in the quality control of mitochondria and in the maintenance of cell homeostasis, we hypothesized that AMBRA1^ActA^-induced mitophagy could be protective in well-known *in vitro* models of PD. We thus decided to test the potential effect of AMBRA1^ActA^ in SH-SY5Y cells treated with 6-OHDA (100 μM, 18 h). In this context, neurotoxicity is induced by blocking activities of mitochondria complexes I and IV (Glinka et al., 1997). In an MTS assay (3-(4,5-dimethylthiazol-2-yl)-5-(3-carboxymethoxyphenyl)-2-(4-sulfophenyl)-2H-tetraz olium) we observed, as expected, a reduction in the metabolic activity of PcDNA3-positive cells following 6-OHDA, when compared to PcDNA3-positive control cells, treated only with the vehicle. By contrast, MTS metabolism was improved in AMBRA1^ActA^-positive cells (Figure 2A). These findings suggest that AMBRA1^ActA^-positive cells are more resistant to neurotoxicity induced by 6-OHDA. To confirm this result, SH-SY5Y cells were transfected with Venus^ActA^ or Myc-AMBRA1^ActA^ or Myc-AMBRA1^ActALIRAA^ for 24 h; cell viability was then measured by means of DAPI nuclear staining, following 6-OHDA treatment. After 6-OHDA treatment, about 80% of Venus^ActA^ or 64% of Myc-AMBRA1^ActALIRAA^ positive cells exhibited condensed or fragmented nuclei, while only 40% of Myc-AMBRA1^ActA^-positive cells showed these apoptotic features (Figure 2B). Therefore, high levels of AMBRA1 at the mitochondria improve the survival of SH-SY5Y cells following 6-OHDA treatment.

To strengthen our finding we next checked, by western-blot analysis, the occurrence of poly (ADP-ribose) polymerase-1 (PARP-1) cleavage, a well-known substrate of caspase-3, in cells over-expressing PcDNA3 or Myc-AMBRA1^ActA^ upon 6-OHDA treatment. As shown in Figure 2C, the cleavage of PARP-1 is reduced in cells overexpressing Myc-AMBRA1^ActA^ compared to cells expressing the control vector (Figure 2C). These results strongly support the existence of a protective effect of AMBRA1^ActA^ against neurotoxicity induced by 6-OHDA treatment in a human neuroblastoma cell line. Since Van Humbeeck et al. (2011) demonstrated that downregulation of AMBRA1 inhibits mitophagy in SH-SY5Y cells, we decided to check whether down-regulation of AMBRA1 expression by RNA intereference (siRNA) could lead to a stronger apoptosis in 6-OHDA-treated cells. As shown in Figure 2D, downregulation of AMBRA1 increases apoptosis, as indicated by increased PARP cleavage, when compared to SiRNA-Ctr positive cells. Moreover, the level of the mitochondrial marker MnSOD does not decrease in a toxic context affecting mitochondria in the absence of AMBRA1 (Supplementary Figure S2A). These results suggest that AMBRA1 plays pro-survival functions in SH-SY5Y cells following 6-OHDA treatment.

These results indicate that AMBRA1^ActA^ confers protection from cell death-induced by 6-OHDA thanks to its mitophagic activity.

### AMBRA1^ActA^-Induced Mitophagy Protects SH-SY5Y Cells From Neurotoxicity Induced by Rotenone

**Figure 3:**
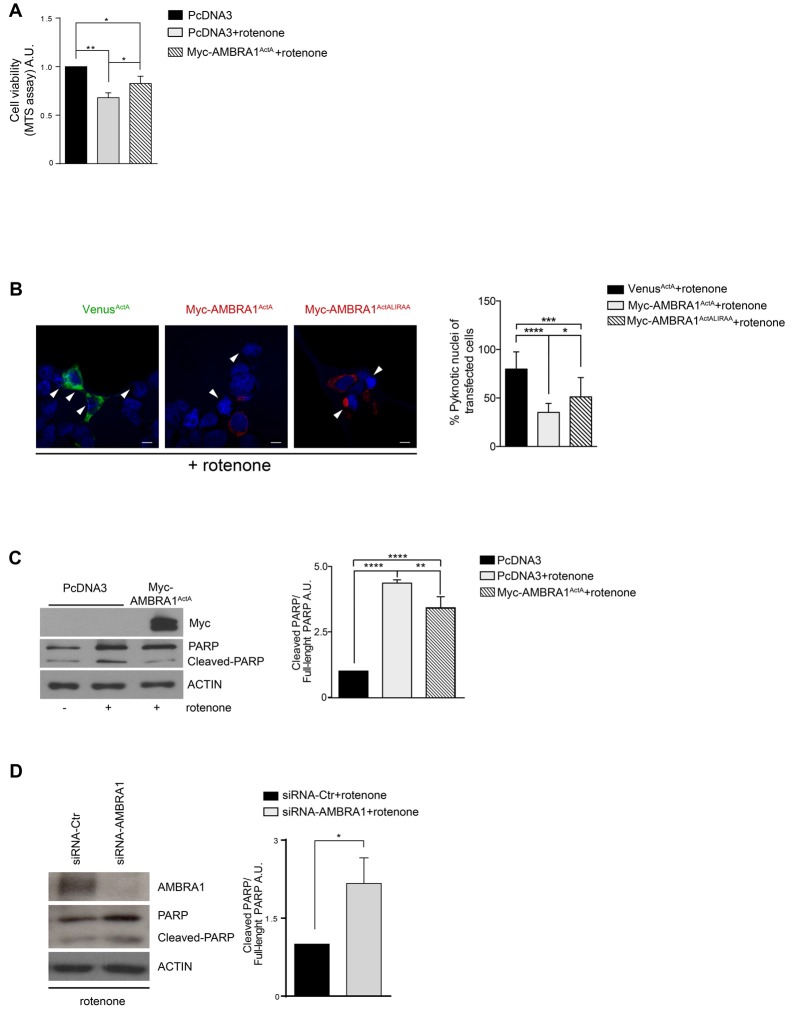
AMBRA1^ActA^ improves vitality of SH-SY5Y cells treated with rotenone. **(A)** SH-SY5Y cells were transfected with vectors coding for PcDNA3 or for Myc-AMBRA1^ActA^ and then treated with rotenone (10 μM). Viable cells were estimated using the MTS assay. Results are expressed as arbitrary unit (A.U.). Each point represents the mean (± SD) of triplicate wells from three independent experiments. Statistical analysis was performed using One-way ANOVA. **P < 0.01. *P < 0.05. **(B)** SH-SY5Y cells were transfected with vectors encoding Venus-ActA or Myc-AMBRA1^ActA^ or Myc- AMBRA1^ActALIRAA^ for 24 h. Six hours after transfection, cells were treated with rotenone (10 μM). After DAPI staining, cells with condensed or fragmented nuclei were scored as pyknotic (arrows indicate pyknotic nuclei). The graph shows the percentage of pyknotic nuclei in transfected cells (± S.D). For each condition, transfected cells were counted in random fields from three independent experiments. Statistical analysis was performed using One-way ANOVA. ****P < 0.0001; ***P < 0.001; *P < 0.05. **(C)** SH-SY5Y cells were transfected with vectors encoding PcDNA3 or Myc-AMBRA1^ActA^ for 24 h. Six hours after transfection, cells were treated with rotenone (10 μM). After extraction of proteins, we performed a western blot analysis using antibodies directed against AMBRA1 (Myc), PARP/Cleaved-PARP and ACTIN. The graph shows the cleaved PARP/full-length PARP *ratio*, resulting as the mean of three independent experiments (± S.D). *n* = 3. One-way ANOVA, *P < 0.05. **(D)** SHSY5Y cells transfected with a siRNA-Ctr or siRNA-AMBRA1 were treated with 6-OHDA for 18 h. Total lysates were subjected to immunoblotting for AMBRA1, PARP and ACTIN antibodies. The graph represents the cleaved PARP/Full-length PARP *ratio* (± S.D). Statistical analysis was performed using One-way ANOVA. *P < 0.05. *n* = 3 independent experiments.

To ascertain that AMBRA1^ActA^ expression was also protective in another ROS-induced dopaminergic cell death model system, we decided to test the effect of AMBRA1^ActA^ in SH-SY5Y cells treated with another “well-known” dopaminergic neurotoxin, rotenone. In this context, neurotoxicity is induced by blocking activity of mitochondria complex I (Glinka et al., 1997). In analogy with 6-OHDA treatment, by using the MTS assay, we observed a reduction in metabolic activity of PcDNA3-positive cells following rotenone, when compared with PcDNA3-positive controls cells treated only with the vehicle. By contrast, MTS metabolism was slightly improved in AMBRA1^ActA^-positive cells (Figure 3A). These findings confirm that AMBRA1^ActA^-positive cells are in general more resistant to neurotoxicity induced by different agents, in this case rotenone. In order to corroborate our results, we transfected SH-SY5Y cells with Venus^ActA^ or Myc-AMBRA1^ActA^ or Myc-AMBRA1^ActALIRAA^ plasmids for 24 h; cell viability was then evaluated by using DAPI nuclear staining upon rotenone treatment. As shown in Figure 3B, AMBRA1^ActA^ expression is sufficient to robustly reduce the number of pyknotic nuclei in transfected individual cells. Therefore, high levels of AMBRA1 at the mitochondria with a marked mitophagy activity (AMBRA1^ActA^) greatly improve survival of SH-SY5Y cells, following both 6-OHDA and rotenone treatments.

Again, by western-blot analysis upon rotenone treatment, we observed an inhibition of PARP-1 cleavage in cells over-expressing Myc-AMBRA1^ActA^ compared to PcDNA3-positive cells (Figure 3C). Altogether, these results strongly support a protective role for AMBRA1^ActA^ in the classical toxic *in vitro* model of PD induced by rotenone. We also checked in this context whether down-regulation of AMBRA1 expression using siRNA was able to trigger a stronger apoptosis following rotenone treatment. As shown in Figure 3D, downregulation of AMBRA1 increases apoptosis, as indicated by increased PARP cleavage. These results suggest that, also upon rotenone treatment of SH-SY5Y cells, endogenous AMBRA1 is involved in a pro-survival pathway. Moreover, SH-SY5Y cells in which AMBRA1 was downregulated through SiRNA, were treated with rotenone in order to analyze the level of the mitochondrial marker MnSOD. In line with the 6-OHDA treatment, we observed no decrease in a toxic context affecting mitochondria in the absence of AMBRA1 (Supplementary Figure S2B).

These results indicate that AMBRA1^ActA^ confers protection from cell death-induced by rotenone thanks to its mitophagic activity.

### AMBRA1^ActA^-Induced Mitophagy Protects SH-SY5Y Cells From Oxidative Stress Generated by Both 6-OHDA and Rotenone

**Figure 4:**
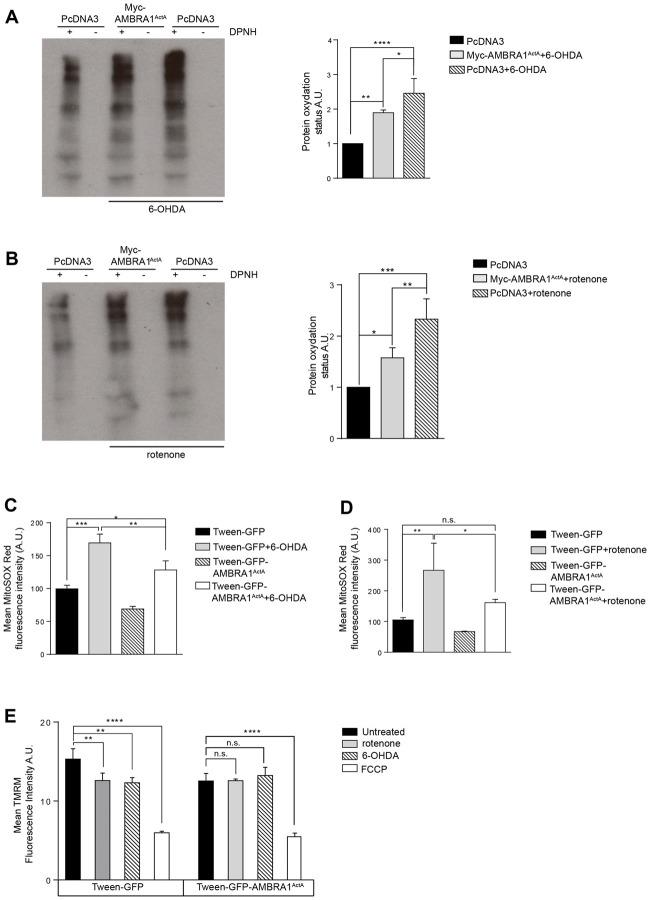
AMBRA1^ActA^ reduces oxidative stress induced by 6-OHDA and rotenone treatments in SH-SY5Y cells. **(A)** Protein carbonylation analysis of SH-SY5Y cells transfected with vectors encoding PcDNA3 or Myc-AMBRA1^ActA^ for 24 h and treated or not with 6-OHDA. The graph showing protein oxidation status (A.U.) results as the mean of three independent experiments (± S.D). Statistical analysis was performed using One-way ANOVA. ****P < 0.0001; **P < 0.01; *P < 0.05. *n* = 3 independent experiments. **(B)** SH-SY5Y cells were transfected as indicated in **(A)** and treated or not with rotenone. The graph shows protein oxydation status (A.U.) and results as the mean of three independent experiments (± S.D). Statistical analysis was performed using One-way ANOVA. ***P < 0.001; **P < 0.01; *P < 0.05. *n* = 3 independent experiments. **(C)** SH-SY5Y cells were transfected with constructs encoding Tween-GFP or Tween-GFP-AMBRA1^ActA^ for 24 h and treated or not with 6-OHDA. Cells were subsequently stained with MitoSOX Red and analyzed by flow cytometry to measure the fluorescence intensity of the dye in GFP positive cells. Data are presented as Mean ± S.D of four independent experiments. Statistical test: One-way ANOVA. *P < 0.05; **P < 0.01; ***P < 0.001. **(D)** SH-SY5Y cells were transfected with constructs encoding Tween-GFP or Tween-GFP-AMBRA1^ActA^ for 24 h and treated or not with rotenone. Cells were subsequently stained with MitoSOX Red and analyzed by flow cytometry to measure the fluorescence intensity of the dye in GFP positive cells. Data are presented as Mean ± S.D of four independent experiments. Statistical test: One-way ANOVA. *P < 0.05; **P < 0.01; ***P < 0.001; n.s.: not statistically significant. **(E)** SH-SY5Y cells were transfected with a plasmid encoding Tween-GFP or Tween-GFP-AMBRA1^ActA^ and then treated or not with rotenone or 6-OHDA or FCCP. Cells were subsequently stained with 5 nM TMRM and analyzed by flow cytometry to measure the fluorescence intensity of the dye in GFP positive cells. Data are presented as Mean ± S.D of three independent experiments. Statistical test: One-way ANOVA. **P < 0.01; ****P < 0.0001; n.s.: not statistically significant.

Although the exact molecular etiology of PD is largely unclear, mitochondrial oxidative stress and accumulated dysfunctional mitochondria are critical factors in the onset of the disease. Thus, we hypothesized that AMBRA1^ActA^-induced mitophagy was able to also protect neuroblastoma cells from neurotoxicity, this being probably due to an anti-oxidative effect. In fact, accumulation of ROS by mitochondria can be reduced by activation of mitochondrial clearance. To test this hypothesis, we transfected SH-SY5Y cells with a vector encoding PcDNA3 or Myc-AMBRA1^ActA^, and treated them with 6-OHDA (100 μM) or rotenone (10 μM) for 18 h. Oxidative modification of proteins by oxygen-free radicals and other reactive species occurs in physiological and pathological processes. As a consequence of the modification, carbonyl groups are introduced into protein side chains by a site-specific mechanism. We thus decided to immunodetect these carbonyl groups, which are hallmarks of protein oxidation status upon 6-OHDA or rotenone treatment in cells overexpressing Myc-AMBRA1^ActA^ or PcDNA3 as a control. As shown in Figures 4A,B, Myc-AMBRA1^ActA^ expression is sufficient to reduce protein carbonylation, upon 6-OHDA or rotenone treatments. To strengthen these results, we analyzed the ROS status through the fluorogenic dye MitoSOX Red coupled to flow cytometry analysis. SH-SY5Y cells were transfected with a bicistronic construct (Tween-GFP-AMBRA1^ActA^) encoding AMBRA1^ActA^ and a cytosolic GFP, or the GFP alone, in order to select cells positive to transfection. Cells were then treated with 6-OHDA and rotenone, as indicated above. As shown in Figures 4C,D, 6-OHDA and rotenone strongly stimulate ROS production, an effect attenuated upon AMBRA1^ActA^ overexpression.

These results indicate that AMBRA1^ActA^ expression reduces oxidative stress induction in SH-SY5Y cells, following induction of neurotoxicity. Next, since the formation of mitochondrial ROS (mtROS) is dependent on ΔΨm (Korshunov et al., 1997), we checked for mitochondrial membrane status using Tetramethylrhodamine, methyl ester (TMRM) in AMBRA1^ActA^ positive cells, following 6-OHDA or rotenone treatments. As shown in Figure 4E, 6-OHDA or rotenone reduce the TMRM signal in control cells, thus confirming an alteration in the mitochondrial membrane potential. By contrast, as previously observed, we found that AMBRA1^ActA^ surrounds depolarized mitochondria and the 6-OHDA or rotenone treatment does not affect mitochondrial membrane potential in AMBRA1^ActA^-transfected cells. Altogether, these data suggest that AMBRA1^ActA^ expression is sufficient to improve mitochondrial membrane status upon neurotoxin treatments.

## Discussion

Our previous study performed on mouse embryonic fibroblasts from *PINK1^−/−^* mice or by using fibroblasts derived from PARKIN and PINK1-mutated PD patients provided evidence that AMBRA1 can mediate mitophagy in the absence of PARKIN or PINK1 activities (Strappazzon et al., 2015). Here, primed by these findings, we decided to investigate the putative therapeutic potential of AMBRA1 to rescue mitochondrial dysfunction in two *in vitro* models of ROS-induced dopaminergic cell death.

Oxidative stress, which can be due to several genetic and environmental factors, is believed to be one of the major mediators of PD pathogenesis. The role of oxidative stress produced by dopamine for neuronal cell survival has been demonstrated in several works (Offen et al., 1996; Gilgun-Sherki et al., 2001). Interestingly, dysfunctional mitochondria are thought to be the predominant source of ROS (Jiang et al., 2016).

In this study, we first decided to mimic PD *in vitro*, by using 6-OHDA, the pro-oxidant derivate of dopamine widely used in ROS-induced dopaminergic cell death model systems. Indeed, 6-OHDA has been shown to increase protein oxidation (accumulation of carbonylated protein), and to increase both caspases-3/7 activity and nuclear fragmentation (Elkon et al., 2002; Hanrott et al., 2006). Interestingly, neurotoxicity of 6-OHDA is significantly attenuated by pre-incubation with catalase, this suggesting that hydrogen peroxide, at least in part, is responsible for cell death in this model. Here we report that AMBRA1^ActA^ expression is sufficient to induce functional mitophagy in neural dopaminergic SH-SY5Y cells. In addition, we found that cell metabolism was improved in the presence of AMBRA1^ActA^ and was associated to apoptosis reduction (reduction of PARP cleavage and occurrence of pyknotic nuclei), following 6-OHDA treatment.

We also investigated the putative beneficial role of AMBRA1^ActA^-mediated mitophagy in a second model of PD by using rotenone. Rotenone is, indeed, a commonly used pesticide, which has been associated with increased risk for PD (Chin-Chan et al., 2015). It is an inhibitor of mitochondrial complex I (Degli Esposti et al., 1993) and it is believed to be a strong ROS producer (McLennan and Degli Esposti, 2000). Systemic chronic exposure of rats to rotenone causes a selective degeneration of dopaminergic neurons in the substantia nigra (Betarbet et al., 2000; Sherer et al., 2003). *In vitro*, rotenone induces cytotoxicity via ROS-induced oxidative stress and mitochondria-mediated apoptosis involving p53, Bax/Bcl-2, and caspase-3 (Siddiqui et al., 2013). Again, by transfecting AMBRA1^ActA^ in SH-SY5Y cells treated with rotenone, we were able to delay apoptosis of human neuroblastoma cells.

These data are of the highest interest, since we were able to show that AMBRA1-mediated mitophagy is cell protective in two independent models of ROS-induced dopaminergic cell death (by 6-OHDA or rotenone). We thus here propose a model by which AMBRA1 expression at the mitochondria is sufficient to reduce ROS accumulation generated by neurotoxin (6-OHDA or rotenone) exposure. This protective effect is due to AMBRA1-mediated mitophagy that favors elimination of dysfunctional mitochondria, thus preventing cells from undergoing death. Indeed, our results strongly suggest that AMBRA1 is a promising target for limiting ROS production and toxin-induced cell death. Of note, it has been recently demonstrated that the mitochondria autophagy receptor Nip3-like protein X, named NIX [also known as BCL2/adenovirus E1B 19 kDa interacting protein 3-like (BNIP3L)] can restore mitophagy and mitochondrial function in fibroblasts from PINK1-PARKIN-mutated PD Patients (Koentjoro et al., 2017). Since NIX, similar to AMBRA1, induces alternative mitophagy (PINK1- and PARKIN-free), it would be of the highest interest to test in this context whether NIX-mediated mitophagy can rescue cell death following 6-OHDA or rotenone treatments in human neuroblastoma cell lines. Interestingly, AMBRA1 presents several common features with NIX such as: (1) a mitochondrial localization (Strappazzon et al., 2011); an LC3-Interacting Region (Strappazzon et al., 2015); a BH3-Like domain (Strappazzon et al., 2016). We could thus hypothesize that AMBRA1 and NIX work in synergy or by separate pathways that may compensate each other. Under physiological conditions, it has been demonstrated that NIX mediates mitophagy in erythrocytes. In particular, *Nix* knockout mice exhibit a reduced number of mature erythrocytes and show defects in mitochondrial clearance (Sandoval et al., 2008). To better understand the function of AMBRA1-alternative mitophagy *in vivo*, it will also be of great interest, and in line with the role of NIX, to examine *Ambra1* deficient mice in the hematopoietic system.

Further studies are needed to better understand the mechanisms by which AMBRA1-mediated mitophagy is initiated and to determine whether AMBRA1-mediated mitophagy could be pharmacologically induced *in vivo* in order to ameliorate the mitochondrial defects observed in human dopaminergic neurons in models of PD.

Finally, we reported here that AMBRA1 is able to favor the survival of SH-SY5Y cells following neurotoxic stimuli by attenuating oxidative stress. Interestingly, the endogenous antioxidant-reduced glutathione has been shown to improve cell viability following 6-OHDA treatment, this indicating that potent brain-penetrating antioxidants might act to slow down PD progression. We are now proposing AMBRA1 as a novel target to reduce oxidative stress; indeed, this molecule could be modulated in the future as a pharmacological target in brains of PD patients or other patients with pathologies associated to oxidative stress.

In sum, our data strongly suggest that alternative mitophagy mediated by AMBRA1 can exert protective effects against PD-related neuronal injury, through inhibiting oxidative stress and mitochondrial dysfunction.

## Author Contributions

AD performed the majority of the experiments planned together with FS and FC. PD performed cloning. PD, LS and SC performed FACS analysis. AD, FS and FC wrote the manuscript.

## Conflict of Interest Statement

The authors declare that the research was conducted in the absence of any commercial or financial relationships that could be construed as a potential conflict of interest.

## Supplementary Material

FIGURE S1 The recombinant protein AMBRA1^ActA^ induces mitophagy in SH-SY5Y. SH-SY5Y cells transfected with PcDNA3 (empty vector) and with plasmids encoding the recombinant proteins Venus^ActA^, Myc-AMBRA1^ActA^, or Myc-AMBRA1^ActALIRAA^ and the wild-type protein Myc-AMBRA1 were fixed and stained for anti-HSP60 (red) and DAPI (blue). Scale bar, 6 μm. The graph shows the HSP60 fluorescence intensity per cell (±S.D). *n* = 3 independent experiments. Statistical analysis was performed by using One-way ANOVA. *****P* < 0.0001; **P* < 0.05.

FIGURE S2 *Endogenous* AMBRA1 downregulation produces a delay in mitochondrial clearance in 6-OHDA- or rotenone-treated cells. **(A)** SH-SY5Y cells were transfected with siRNA-Ctr or a siRNA against AMBRA1 and treated with 6-OHDA for 18 h. Total lysates were subjected to western blotting analysis for the indicated antibodies. **(B)** Total lysate of SH-SY5Y cells downregulated for AMBRA1, through siRNA-AMBRA1 transfection were treated with rotenone for 18 h and then analyzed by western blotting analysis looking to mitochondrial marker MnSOD level.
