# Supplementary figures and images for: AMBRA1-Mediated Mitophagy Counteracts Oxidative Stress and Apoptosis Induced by Neurotoxicity in Human Neuroblastoma SH-SY5Y Cells
Source: Front Cell Neurosci. 2018 Apr 18;12:92. doi: 10.3389/fncel.2018.00092 (PMC5932353; doi:10.3389/fncel.2018.00092)

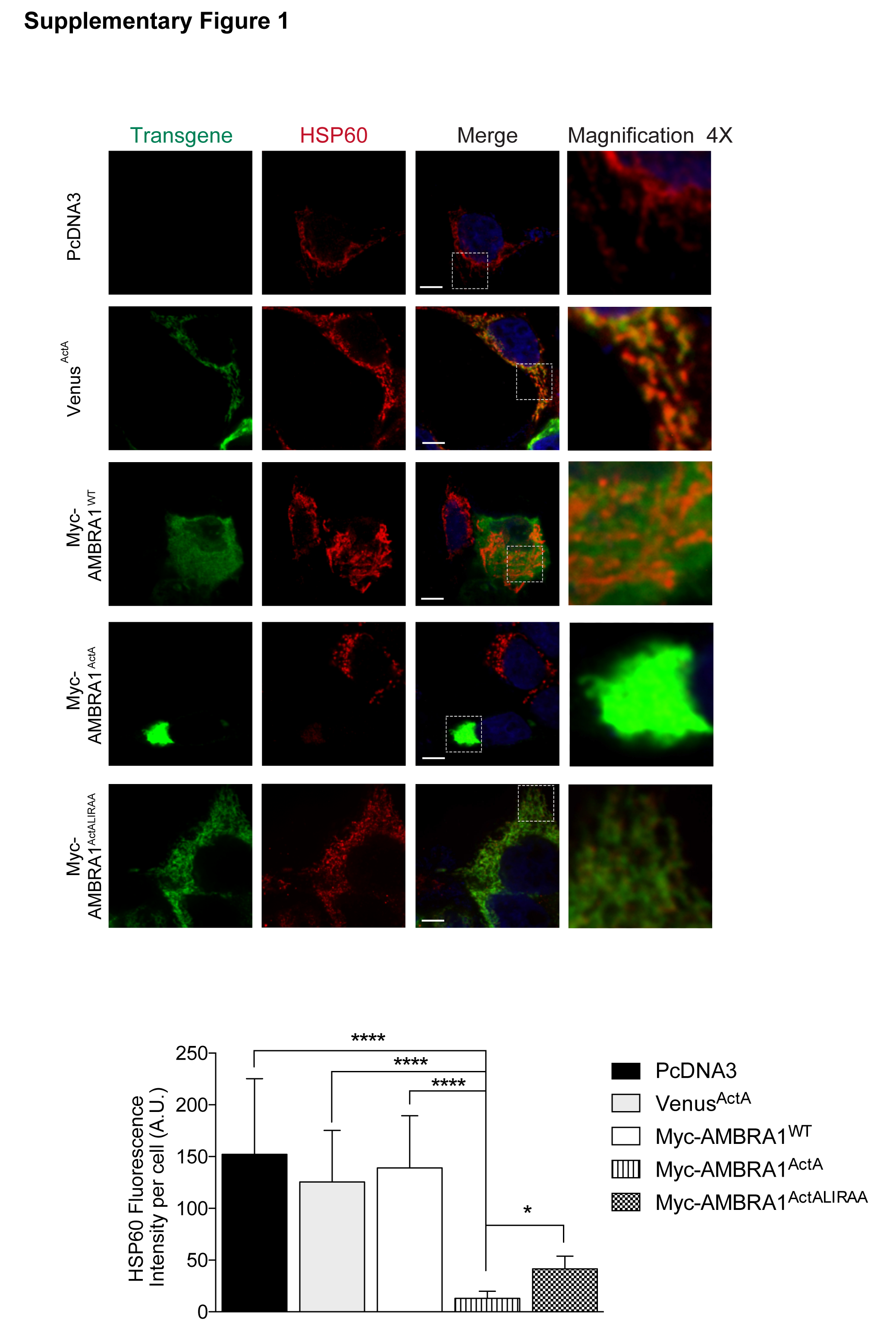

Supplement: FIGURE S1 — The recombinant protein AMBRA1ActA induces mitophagy in SH-SY5Y. SH-SY5Y cells transfected with PcDNA3 (empty vector) and with plasmids encoding the recombinant proteins VenusActA, Myc-AMBRA1ActA, or Myc-AMBRA1ActALIRAA and the wild-type protein Myc-AMBRA1 were fixed and stained for anti-HSP60 (red) and DAPI (blue). Scale bar, 6 μm. The graph shows the HSP60 fluorescence intensity per cell (±S.D). n = 3 independent experiments. Statistical analysis was performed by using One-way ANOVA. ****P < 0.0001; *P < 0.05. [file Image_1.tif]

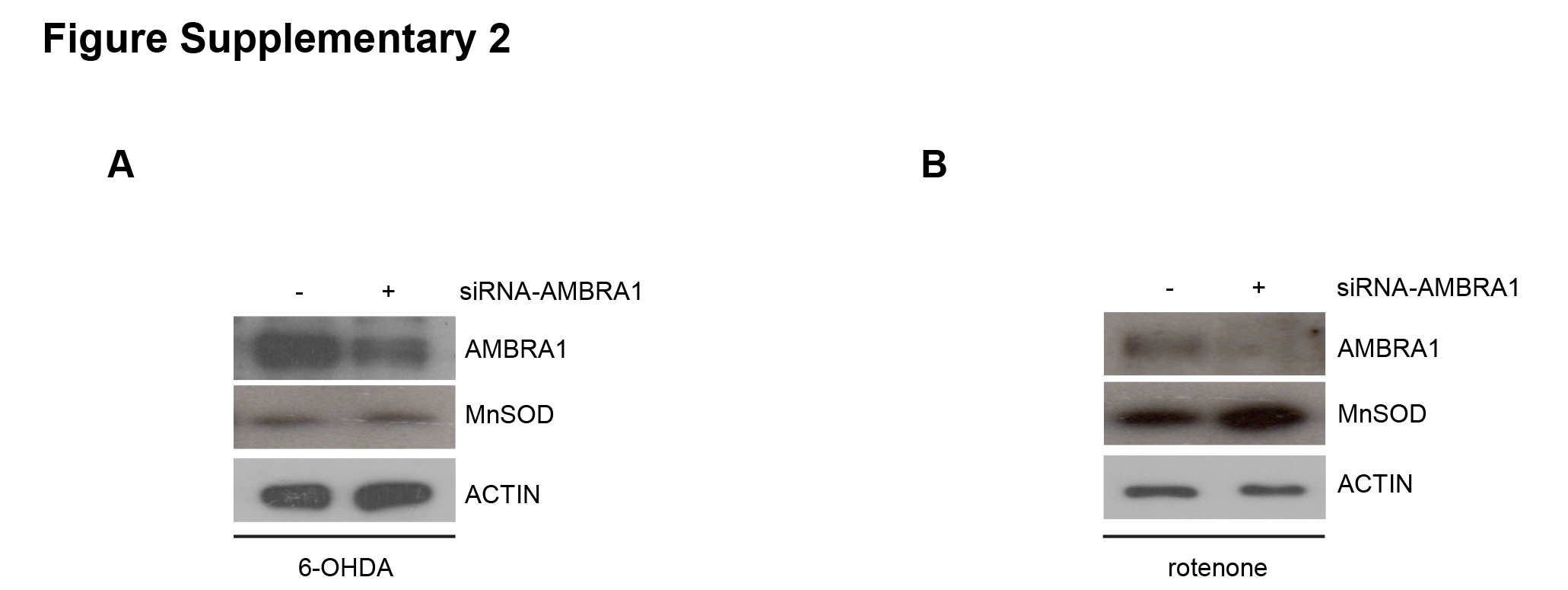

Supplement: FIGURE S2 — Endogenous AMBRA1 downregulation produces a delay in mitochondrial clearance in 6-OHDA- or rotenone-treated cells. (A) SH-SY5Y cells were transfected with siRNA-Ctr or a siRNA against AMBRA1 and treated with 6-OHDA for 18 h. Total lysates were subjected to western blotting analysis for the indicated antibodies. (B) Total lysate of SH-SY5Y cells downregulated for AMBRA1, through siRNA-AMBRA1 transfection were treated with rotenone for 18 h and then analyzed by western blotting analysis looking to mitochondrial marker MnSOD level. [file Image_2.tif]
